# Periscope

**Published:** 1868-10

**Authors:** 


### PERISCOPE.

### OULACHAN OIL AS A SUBSTITUTE FOR COD-LIVER OIL.

The following valuable article has been recently published in the *Pharmaceutical Journal and Transactions*, (London, June, 1868,) by the distinguished botanist and zoölogist, ROBERT BROWN. As our possessions have lately been extended on the north-west coast, it has peculiar commercial and therapeutical interest, and we copy it therefore at length:—

"The fish which forms the subject of this communication may, if we consider its importance to the Indians, or the still more useful purposes to which both the fish itself and its oil might be applied, without fear of contradiction be ranked as one of the most valuable products of the western shores of America. Many of the earlier fur-traders and adventurers refer to it in enthusiastic terms under its Chinook name of *Oulachan* or *Eulachon*,* and give accounts of its abundance in the Columbia River early in this century. All readers of WASHINGTON IRVING'S charming 'Astoria,' cannot fail to remember his description of it. It belongs to the family *Salmonidæ*, and is usually classed in Gerard's genus *Thaleicthys*, but as I believe that that genus is separated from the older one of *Osmerus* on very insufficient grounds, I have preferred to designate it as *Osmerus pacificus*. The synonymy and specific characters will therefore stand as follows:—

OSMERUS PACIFICUS (*Salmo* (*Mallotus*) *pacificus*), Richardson Fauna Boreali-Americana; *Thaleicthys Stevensi*, GERARD, Gen. Rep. on Fishes; *Thaleicthys pacificus*, "Grd." COOPER and SUCKLEY Natural History of Washington Territory, Plate LXXV. figs. 1–4; *Osmerus pacificus*, (Rich.) AYRES, Proceedings Cal. Acad. Nat. Science, ii. 64. Head subconical and pointed. Mouth large; posterior extremity of maxillar bone extending to a vertical line drawn posteriorly to the orbit. Eye rather small. Adipose fin placed opposite the posterior portion of the anal, which is rather elongated. The insertion of the ventral fins is situated considerably in advance of the anterior margin of the dorsal. Scales moderate, subelliptical. Dorsal region grayish-olive; middle of flank yellowish-orange,

*Ross Cox calls it "the sweet little anchovy" ('The Columbia River,' etc., vol. i. p. 105.) It is also spelt *hoolakan* and Ulichan. Alexander Ross calls it the "fathom fish," because strung on threads in their dried condition, they were sold by the fathom ('Adventures of First Settlers on the Columbia River,' p. 94).

dotted with black; belly yellowish, unicolor; upper sides and surface of head grayish; fins unicolor.

2. The *Oulachan* or *Eulachon* is a small, delicate-looking fish, about the size of a smelt, and not unlike it, semipellucid, and with fine scales. On or about the 24th of March,—at nearly the same time each year,—it enters the northern rivers, and the southern ones a little later. It was once abundant in the Columbia, but that stream being now disturbed by the traffic of steamers, it is only now in exceptional years that they are caught there in any quantity. In Fraser River, and in most of the rivers on the coast of British Columbia, they are still found at that season (March) in greater or smaller quantities; but it is in the Naas River, falling into the Pacific, in lat. 54° 40′ N., that the Eulachon is found in the greatest quantities, and it is to its capture in that stream that these notes chiefly relate. The fish comes up from the sea into the fresh water for the purpose of spawning, but, unlike most of its allies,—the salmon proper,—on the coast, returns to the sea again, and is not seen until the following year. During that season they swarm in inconceivable shoals, and I can well believe that the Indians indulge in no hyperbole when I have heard them say that their canoes have been lifted in the water by the countless swarms of fishes. Their arrival is at once heralded by flocks of *Laridæ* and other marine birds swooping down to seize upon them, and during the whole of the fishing season the screams of the gulls vie with the shouts of the Indian fishers.

3. By long custom made and provided for, northern tribes have a vested right of fishing the Eulachon on the banks of the Naas, and certain other equally numerous and powerful tribes are prohibited from enjoying this privilege, and are compelled to buy their oil from their more fortunate neighbors. Accordingly, some days before the expected advent of the fish in the river, the Indians assemble from far and near to the number of several thousands, in order that they may take up their proper camping-grounds on the banks. Men, women, and children come,—it is the herring-fishing of the Indians, and all can be employed. A general holiday prevails, and tribes vie with tribes, families with families, in dress and feasting, and show their joyousness in a thousand different ways. Families who have not met for twelve months now meet, and the Eulachon, or *Yghuh* (almost unspellable and certainly unpronounceable), fishing is looked forward to from one year's end to the other as a time for gossiping, courting, and general merrymaking. In a few days, however, the fish begin to make their appear-

39

ance, and now all are on the alert, and all idling is at an end. The first shoal, as I have said, come into the river, from the 24th to the 27th of March, and stay three days. These are so exceedingly fat that they cannot be cooked in a pan, for they will "blaze up" like a mass of oil. Out of these the best portion of the oil is made. In about three days these begin to disappear, and are succeeded by a second shoal, not so large or so fat, and these again in a day or two by the third and last shoal, which is poorer, and are dried for winter use, being sufficiently free from oil to permit of this. So fat are these last even, that if lighted during the dry state, they will burn like a candle, and are often used as such by the natives, hence they are sometimes called the "candle-fish." The river during the time of fishing presents a busy scene, covered with canoes sweeping the fish in, while others filled are landing and being unloaded by the women and children, again wildly to rush back to share in the harvest. Ashore the scene is not less vivid. Fires are blazing and pots boiling, and boxes being filled with the oil, while in and around and all over, prevails an amount of unctuousness indescribable,—a greasiness of which it is impossible to conjure up the faintest idea! The fish are chiefly taken by nets (in the Naas), but myriads get washed ashore and are caught by the old women and children and kept as their perquisite. In Fraser River they are principally captured by means of a flattened cedar pole, the edges of which for a couple of feet or so near the end being set with sharp teeth or nails, which act like so many spear-points. The Indian, standing in his canoe, sweeps this through the water, and so numerous are they that there is no fear but that a number will be impaled on the points. These are swept behind him into the canoe as a mower uses a scythe, until the canoe is full. Herrings and shoals of all other small fishes are caught likewise in this ingenious mode. Besides those kept for drying or from which the oil is made, vast quantities are used in the fresh state for food, and the sudden arrival of fish, occurring generally just at a time when the Indians' winter stores are nearly finished and they are rather pressed for food, the plethora often proves fatal by producing surfeit.

4. The oil is obtained by putting the fish into water in boxes —generally hollowed out of a solid block of cedar (*Thuga gigantea*, Nutt., *T. Menziesii*, Dougl.), or so closely made as to be water-tight—and then throwing in red-hot stones. This ingenious method of boiling is practised by all the Indians on the north-west coast of America. The oil is then skimmed off the surface and set aside to cool. The oil is never made by suspending iron vessels (after the more familiar manner of the whites) over the fire, for in that case the fishes would be destroyed, and it would be difficult to separate the broken fragments from the oil. The quality, however, greatly depends upon the care employed, and the amount of heat used to extract the oil from the fatty tissues of the fish. An inferior description is also made by squeezing the fishes out of which the finer oil has already been extracted in the method described, in a cloth against a board.* Properly prepared, the oil is at a temperature of 60° Fahr., amber-colored and liquid. At a lower temperature it becomes thick and opaque, increasing in solidity according to the degree of cold; in this state it is whitish in color, and resembles soft lard. The northern tribe keep it in boxes of their own making, but the more southern Indians—such as the Quăkwolths, at Fort Rupert (lat. 50° 42′ 36″ N., long. 127° 25′ 07″ W.)—preserve it in bottles, made out of the stem of the giant seaweed, *Macrocystis pyrifera*, Ag., squeezing out a little, when required, as a painter does his colors out of the tinfoil tubes.

5. The fish, cooked fresh, is most delicious, and, when salted, is also a very palatable article of food, and held in much re-

* I have given the general rationale of the process of manufacture. There are, however, various superstitions connected with the *oulachan* (as with everything else the Indian has to do with), which entail various minute ceremonies. Mr. William Duncan, the excellent missionary at Metlakatah, thus refers to it in a letter addressed to the Church Missionary Society:—"...The process (of extraction) is as follows: Make a large fire; place three or four heaps of stones as big as your hand in it; while these are heating, fill a few baskets with rather stale fish, and get a tub of water into the house. When the stones are red-hot, bring a deep box, about eighteen inches square, near the fire, and put about half a gallon of the fish into it and as much fresh water, then three or four hot stones, using wooden tongs. Repeat the doses again, then stir up the whole. Repeat them again, stir again; take out the cold stones and place them in the fire. Proceed in this way till the box is nearly full, then let the whole cool, and commence skimming off the grease. While this is cooking prepare another box in the same way. In doing the third, use, instead of fresh water, the liquid from the first box. On coming to the refuse of the boiled fish in the box, which is still pretty warm, let it be put into a rough willow basket, then let an old woman, for the purpose of squeezing the liquid from it, lay it on a wooden grate, sufficiently elevated to let a wooden box stand under; then let her lay her naked chest on it, and press with all her weight. On no account must a male undertake to do this. Cast what remains in the basket, anywhere near the house, but take the liquid just saved and use it over again instead of fresh water. The refuse must be allowed to accumulate, and though it will soon become putrid and change into a heap of maggots, and give out a smell almost unendurable, it must not be removed. The filth contracted by those engaged in the work, must not be washed off until all is over; that is, till all the fish are boiled, and this will take about two or three weeks. All these plans must be carried out without any addition or change, otherwise the fish will be *ashamed* (the Indians think), and perhaps never come back again."

quest among the Hudson Bay Company's traders and other old residents on the coast. The Indians dry vast numbers for winter use, and carry them with them in strings, during their annual migrations south, and for sale to other tribes who come to purchase them as well as oil. The *Tsimpsheans* say that the Naas river clothes them and the Skeena river feeds them, because the *Hydahs*, from the Queen Charlotte Islands, and other tribes who are prohibited from fishing for the *Oulachan* in the Naas, come and purchase the oil from them, paying blankets for it, while the salmon of the Skeena supplies them with abundant supplies of food. I cannot but think that these fish would form a most valuable and lucrative article of commerce either in the salt or dried condition, and that in either of these forms, or preserved in ice, or in their own olive oil, like sardines, they would command a ready market, especially in the Roman Catholic countries along the Pacific coast, in China, and even in Europe, and the Atlantic States of America. A small joint stock company was indeed formed in Victoria, in 1864, for that purpose, but failed for want of capital and in ignorance of the habits of the fish. Before they could get their affairs settled to start north, the season was past, and nothing further was ever done. The Indians, no doubt, declare that no white man shall ever cast a net into the Naas, but independently of this somewhat futile threat, supplies could be purchased from the Indians to almost any amount, and, if sufficient inducement were held out to them, the present catch could very easily be increased tenfold.

6. The oil is of even greater value than the fish itself, as usually seen in the opaque lard-like condition, and after having undergone no other preparation than the rough *trying out* just described, its taste is not unpleasant, and the odor by no means disagreeable. Even in this condition, it has been used by the whites for culinary purposes, and the Indians use it in all their meals, much after the same way as we do butter, using it also as a sauce to their dried salmon. So fond are they of it, and so essential to their health is it (as I shall presently refer to), that the Hydahs and other tribes, as I have already said, come over to purchase it eagerly, and the Hydahs, Stekins, Tsimpsheans, and other northern tribes that winter in Victoria and Puget Sound, will come on board the Metlakathlah mission schooner to purchase it. They complain of the price, but still cannot do without it. An old Tsimpshean once said to me, "I can buy beef and bread cheaper, but my heart never feels good until I have got this grease. There are just two sweet things in food—*rum* and oulachan oil!" However much we may be inclined, from a civilized stand-point of view, to doubt the soundness of this summation of a lifetime's experience, there is no doubt that this oil, both in an edible and *medicinal* light, is of the utmost value. It is the latter property which the readers of the present article will be most interested in, and which I desire most earnestly to press upon their attention. Its effects on phthisical patients are most wonderful, and, from the moist climate of the northern portions of the Pacific coast, the natives are very subject to phthisis, hæmoptysis, and other forms of pulmonary disease. As it is, many die annually of these complaints, and I believe that I only speak the opinion of all who know these people, or who have thought over the subject, that were it not for this oulachan oil, these northern tribes, once so powerful, and still so courageous, intelligent, and physically fine, would be decimated, and already enfeebled in constitution through vices learnt from the whites, their extermination would soon be *un fait accompli.* It relieves violent coughs in a most remarkable manner, and equally conduces to the accumulation of flesh. In a word, it has all the properties of cod-liver oil and other fish oils in an intensified degree, without their nauseous taste—a taste which is found even in the best and most carefully prepared oils, and prohibits many availing themselves of their valuable qualities. I have known delicate ladies who would have vomited at the smell of the ordinary cod-liver oil, put the bottle of oulachan oil (slightly heated, in order to liquefy it) to their mouths, and drink it without the smallest nausea! If the oil thus rudely prepared by the natives be so little unpalatable, I doubt not but that if it underwent the usual refining processes of the chemist, that it might be produced perfectly tasteless. The old fur traders on the coast everywhere use it in pulmonary diseases, and even send supplies of it into the interior, for the use of friends residing there. It is looked upon almost as a specific, and the few boxes which the Hudson Bay Company's trading vessel brings down on her annual spring voyage (not as an article of commerce, but for the accommodation of friends,) are generally bespoke long before. The medical officers of the Company have long preferred prescribing it to cod-liver oil, both in their own families and in general practice. One of these gentlemen, whose great intelligence and long experience entitle his opinions to every respect,* enter_

* I believe I am at liberty to mention his name. The Honorable JOHN SEBASTIAN HOLMECKEN, Chief Trader and Surgeon H. B. C., Member of the Legislative Council of British Columbia, and formerly Speaker of the Legislative Assembly of Vancouver Island.

tains very similar views to those I have advocated, and I have, moreover, heard him attribute the health and even the existance of the Indians during their exposed life in a *hyperpluviose* climate like that of Fort Simpson and north to Sitka, to the use of oulachan oil. In the course of my journeys into the interior of Oregon and elsewhere, I had occasion to recommend and procure some for friends troubled with phthisical complaints, and in every instance I have heard its merits extolled to the highest degree.

7. The object of this paper has been to draw the attention of pharmaceutists to this oil, and with a view to its being tried in a medicinal and commercial way. In 1864, some specimens were sent to England, and became rancid before arriving, though even in that condition they were valued at the rate of £40 per ton; but I am not aware that it has ever yet been tried in European medicinal practice. I have no doubt that if efforts were made to procure a sufficient quantity to give it a proper trial at the hands of physicians, whose opinions would carry weight with them, the *Oleum Osmeri* would prove a useful addition to our animal Materia Medica, as auxiliary to, or substitute for, the better known and justly esteemed *Oleum Jecoris Aselli* of the Pharmacopœia."

